# Perspectives and Experiences of Patient-Led Melanoma Surveillance Using Digital Technologies From Clinicians Involved in the MEL-SELF Pilot Randomized Controlled Trial: Qualitative Interview Study

**DOI:** 10.2196/40623

**Published:** 2022-12-20

**Authors:** Dorothy Drabarek, Emily Habgood, Deonna Ackermann, Jolyn Hersch, Monika Janda, Rachael L Morton, Pascale Guitera, H Peter Soyer, Helena Collgros, Anne E Cust, Robyn PM Saw, Jon Emery, Victoria Mar, Mbathio Dieng, Anthony Azzi, Alister Lilleyman, Katy JL Bell

**Affiliations:** 1 School of Public Health University of Sydney Sydney Australia; 2 Centre for Cancer Research Department of General Practice University of Melbourne Melbourne Australia; 3 Centre for Health Services Research University of Queensland Brisbane Australia; 4 National Health and Medical Research Council Clinical Trials Centre University of Sydney Sydney Australia; 5 Melanoma Institute Australia University of Sydney Sydney Australia; 6 Sydney Melanoma Diagnostic Centre Royal Prince Alfred Hospital Sydney Australia; 7 Sydney Medical School Faculty of Medicine and Health University of Sydney Sydney Australia; 8 Frazer Institute University of Queensland Dermatology Research Centre Brisbane Australia; 9 Dermatology Department Princess Alexandra Hospital Brisbane Australia; 10 The Daffodil Centre University of Sydney Sydney Australia; 11 Melanoma Unit Royal Prince Alfred Hospital Sydney Australia; 12 School of Public Health and Preventive Medicine Monash University Melbourne Australia; 13 Newcastle Skin Check Newcastle Australia; 14 School of Medicine University of Queensland Brisbane Australia

**Keywords:** melanoma, self-surveillance, teledermatology, teledermoscopy, mHealth, high-value care, digital technologies, surveillance, lesion, clinicians, care, mobile, technology, skin

## Abstract

**Background:**

The growing number of melanoma patients who need long-term surveillance increasingly exceeds the capacity of the dermatology workforce, particularly outside of metropolitan areas. Digital technologies that enable patients to perform skin self-examination and send dermoscopic images of lesions of concern to a dermatologist (mobile teledermoscopy) are a potential solution. If these technologies and the remote delivery of melanoma surveillance are to be incorporated into routine clinical practice, they need to be accepted by clinicians providing melanoma care, such as dermatologists and general practitioners (GPs).

**Objective:**

This study aimed to explore perceptions of potential benefits and harms of mobile teledermoscopy, as well as experiences with this technology, among clinicians participating in a pilot randomized controlled trial (RCT) of patient-led melanoma surveillance.

**Methods:**

This qualitative study was nested within a pilot RCT conducted at dermatologist and skin specialist GP–led melanoma clinics in New South Wales, Australia. We conducted semistructured interviews with 8 of the total 11 clinicians who were involved in the trial, including 4 dermatologists (3 provided teledermatology, 2 were treating clinicians), 1 surgical oncologist, and 3 GPs with qualifications in skin cancer screening (the remaining 3 GPs declined an interview). Thematic analysis was used to analyze the data with reference to the concepts of “medical overuse” and “high-value care.”

**Results:**

Clinicians identified several potential benefits, including increased access to dermatology services, earlier detection of melanomas, reassurance for patients between scheduled visits, and a reduction in unnecessary clinic visits. However, they also identified some potential concerns regarding the use of the technology and remote monitoring that could result in diagnostic uncertainty. These included poor image quality, difficulty making assessments from a 2D digital image (even if good quality), insufficient clinical history provided, and concern that suspicious lesions may have been missed by the patient. Clinicians thought that uncertainty arising from these concerns, together with perceived potential medicolegal consequences from missing a diagnosis, might lead to increases in unnecessary clinic visits and procedures. Strategies suggested for achieving high-value care included managing clinical uncertainty to decrease the potential for medical overuse and ensuring optimal placement of patient-led teledermoscopy within existing clinical care pathways to increase the potential for benefits.

**Conclusions:**

Clinicians were enthusiastic about the potential and experienced benefits of mobile teledermoscopy; however, managing clinical uncertainty will be necessary to achieve these benefits in clinical care outside of trial contexts and minimize potential harms from medical overuse.

**Trial Registration:**

Australian and New Zealand Clinical Trials Registry ACTRN12616001716459; https://anzctr.org.au/Trial/Registration/TrialReview.aspx?id=371865

## Introduction

The incidence of cutaneous melanoma continues to increase globally [1,2]. In Australia, a country with high melanoma burden [3] and an aging population [4], this continued increase is largely driven by the increased diagnosis of stage 0-2 localized melanoma, as defined by the American Joint Committee on Cancer (AJCC). After excision of the localized melanoma, patients require long-term clinical surveillance, as they are at high risk of developing a recurrent or a subsequent new primary melanoma [5]. Increasing demand for such dermatology services, a shortage of dermatologists [6], and the proliferation of mobile technologies have prompted consideration of changes to traditional face-to-face modes of clinical surveillance [7]. The COVID-19 pandemic has increased the adoption of store-and-forward or real-time video teledermatology [8], with increased use of “virtual melanoma checks” for triaging whether a biopsy or face-to-face review is needed [9]. Additionally, as many melanomas are initially detected by the patient themselves or a family member [10], patient-performed teledermatology (including teledermoscopy) among people with a personal history of melanoma may allow the early detection of a subsequent melanoma [11]. Teledermatology could both increase patient access to a dermatological opinion and reduce the need for routinely scheduled clinic visits. This may especially benefit patients living in rural and remote areas [12], reduce the burden on the health care system, and free up clinician time [13].

Teledermatology smartphone apps that allow patients to send macroscopic images of concerning lesions to skin specialists have become more readily available to consumers since 2012 [14,15]. Commercial teledermatology services offer a store-and-forward modality of teledermatology as part of remote service delivery models where there is no prior patient-doctor relationship and without referral [14-17]. More recently, store-and-forward teledermoscopy has become available in clinical trial settings, whereby patients take dermoscopic images of concerning lesions using a mobile dermatoscope attached to their smartphone camera and transmit these securely to a dermatologist via a smartphone app [18-21]. If shown to be safe, cost-effective, and acceptable to patients, acceptance by teledermatologists assessing images and by treating doctors is also needed before this new model of care is adopted into routine practice [7,22]. Clinicians’ acceptance is a key factor in the adoption and long-term use of digital technologies in clinical practice [23,24].

We recently conducted the MEL-SELF pilot randomized controlled trial (RCT) of patient-led surveillance using a mobile dermatoscope and app in addition to usual care (intervention) compared to clinician-led surveillance (usual care). We found that patient-led surveillance including teledermoscopy may be a useful addition to routinely scheduled clinic visits [11,25]. This report presents findings from a nested qualitative study conducted with the trial’s teledermatologists, treating dermatologists, and treating skin specialist general practitioners (GPs), referred to collectively as clinicians. We undertook interviews to explore clinicians’ views on teledermatology and patient-conducted teledermoscopy and their experiences using these during the pilot trial.

## Methods

### MEL-SELF Pilot Trial

The MEL-SELF pilot trial ran from November 2018 to January 2020, and a detailed report of the study findings has been published [11]. A total of 100 patients previously treated for melanoma were recruited from specialist-led and GP-led private clinics in Sydney and Newcastle, New South Wales, Australia, and randomized to control (n=51, 51%) or intervention (n=49, 49%). Three treating dermatologists and 6 skin specialist GPs recruited patients, provided routine skin checks, and reviewed lesions as prompted by the intervention, while images submitted from patients by teledermatology were sent to and reviewed by 3 dermatologists located in Sydney, Brisbane, and Melbourne (1 of whom also recruited patients and provided their ongoing care). The model of teledermatology in the MEL-SELF pilot trial was one in which the teledermatologist was not the patient’s treating doctor. Treating doctors did not assess images submitted by teledermatology.

Patients in the intervention group were guided on skin self-examination (SSE) through online videos [26] and were provided with a mobile dermatoscope and smartphone app. Patients took dermoscopic images and submitted these for teledermatology review, together with self-reported history (ie, lesion location, history of change, melanoma history of themselves and family, number of moles, skin type, and patient age). If the teledermatologist assessed the lesion as suspicious for melanoma and recommended urgent clinical review in their report, the patient made a fast-tracked, unscheduled appointment with their treating doctor (facilitated by research staff). Teledermatology technologies were provided by MetaOptima Technology Inc (Vancouver, Canada) [27], including a mobile dermatoscope (MoleScope I) that integrates with MoleScope (a smartphone-based skin imaging app) [28] and DermEngine (a digital software system that facilitates the capture, storage, communication, and analysis of skin images by dermatologists) [29].

Clinicians were eligible to participate in this qualitative study if they were involved in screening and recruiting patients for the MEL-SELF pilot RCT and/or reading submitted images and providing reports via teledermatology.

### Ethics Approval

This study was approved by the Human Research Ethics Committees at the University of Sydney (X15-0445) and the Royal Prince Alfred Hospital (HREC/15/RPAH/593). All participants provided informed consent. The design, conduct, and reporting of this study follow the SRQR (Standards for Reporting Qualitative Research) guidelines [30].

### Data Collection and Analysis

An interview topic guide was developed by the authors to cover views on and experiences of patient-led melanoma surveillance, teledermatology, patient-performed teledermoscopy, and components of trial implementation relevant to each clinician depending on the role they played (Multimedia Appendix 1). The topic guide was flexible enough to adapt to the specific role of each clinician in the pilot trial, and discussions beyond their experiences in the pilot trial were also encouraged. Semistructured phone interviews were conducted by 1 researcher (author DD) who is trained in qualitative interviewing and was not known to any of the clinicians before the study. Interviews lasted between 19 and 77 minutes, with a median time of 23.5 minutes. Interviews were audio recorded and transcribed verbatim by 2 researchers (authors DD and EH). Identifying details were removed from the data at the transcription stage.

Interview summaries were written immediately following each interview. Discussions between authors DD and EH considered emerging analytical ideas and opportunities to refine the topic guide and approach. Preliminary codes were developed inductively from the interview summaries by DD and EH. Both authors tested these by separately coding 4 transcripts. A coding framework was then agreed upon by DD and EH and applied to the rest of the data. Key themes and interpretations were identified through coding, memo writing, and analytical discussions [31] involving authors DD, EH, JH, and KJLB. Our emerging theoretical framework had clear resonance with 2 concepts pertinent to the assessment, improvement, and prioritization of health care service delivery: high-value care and medical overuse. High-value care refers to necessary health care that is supported by evidence showing it benefits patients [32]. Conversely, medical overuse is unnecessary health care that is unlikely to benefit patients but may cause them harm [33,34]. We used these concepts to further organize and interpret the data. Coding and initial organization of codes were done in NVivo 12 software (QSR International), and a data matrix was exported to Microsoft Excel (IBM Corp) to aid thematic analysis.

## Results

### Sample and Overview

All 11 clinicians who were involved in the MEL-SELF trial were invited to participate in an interview. Three clinicians did not reply to the initial or follow-up invitation email, while 8 clinicians agreed to participate and were interviewed between October and November 2020. These included 6 treating clinicians (2 dermatologists, 1 of whom also provided teledermatology, along with 1 surgical oncologist and 3 skin specialist GPs) and 2 dermatologists who provided teledermatology only. Five clinicians were female and 3 were male. Clinicians’ experience in managing melanoma patients ranged from 15 to 20 years.

All clinicians had experience using teledermatology; however, due to the novelty of patient-led teledermoscopy in the MEL-SELF pilot trial, experience reviewing dermoscopic images taken by patients (especially those who were not under their care) was more limited. Only 1 clinician (a teledermatologist) had extensive experience reporting on dermoscopic images taken by patients in research settings. All had experience providing second-opinion reviews of dermoscopic images taken by clinical staff in both clinical and research settings. Along with their experiences in the pilot trial, clinicians discussed their experiences of teledermatology outside of it, and these accounts were included in the analysis.

**Figure 1:**
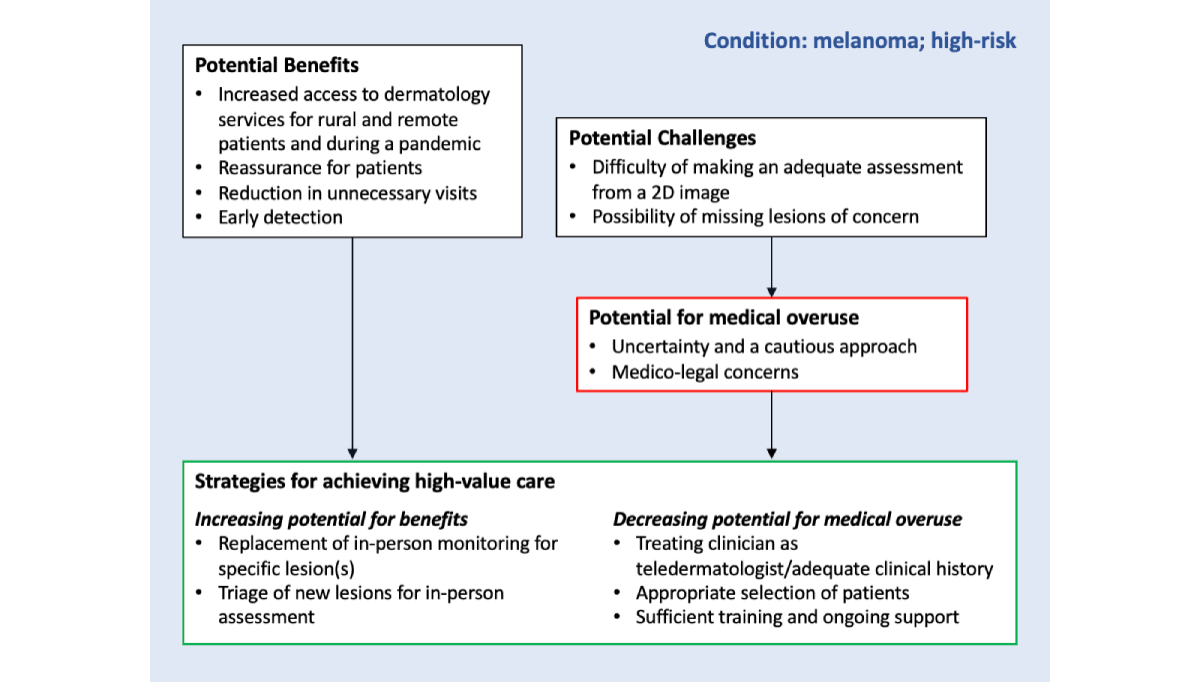
Strategies for patient-led surveillance to achieve high-value care.

The results of the thematic analysis are summarized in Figure 1. For patient-conducted teledermoscopy to deliver high-value care, strategies are needed to both facilitate potential benefits and inhibit potential harms (particularly from medical overuse). Themes and their relationships to each other are explained in the subsequent sections, supported by illustrative quotes. Additional quotes are included in Multimedia Appendix 2.

### Perceived Benefits of Patient-Led Surveillance and Patient-Performed Teledermoscopy

There was an agreement among clinicians that patient-led surveillance is a good idea. Clinicians said that patients should be encouraged to get to know their skin and be educated in self-monitoring, noting, however, that not all patients are interested in doing so. Teledermatology was thought to increase access to dermatology services for rural and remote patients and enable continuity of care for all patients during the COVID-19 pandemic, thereby facilitating the early detection of melanomas. Teledermoscopy was thought to make shared monitoring of particular lesions of concern more convenient for high-risk patients living at a distance from dermatology services. Clinicians said that teledermoscopy enables quick feedback for lesions that the patient thinks are potentially concerning, either facilitating prompt review in the clinic if necessary or otherwise alleviating worry and a potentially long period of anxiety ahead of the next routinely scheduled visit. They also thought it may be useful for monitoring lesions that the clinician thinks are potentially concerning. For this use, remote monitoring was thought to have beneficial potential by reducing the frequency of routinely scheduled clinics for monitoring particular lesions.

### Perceived Challenges and Potential Harms of Teledermoscopy and Teledermatology

#### Making Judgments From a Digital Image

Of the 6 treating clinicians, 5 emphasized that any images taken by patients needed to be of good quality, but in their experience inside and outside of the pilot trial, these were often of suboptimal quality, making clinical decisions difficult. While most of the treating clinicians were not assessing dermoscopic images during the pilot trial, they were able to view the images taken by their own patients. One treating clinician commented about the poor quality of dermoscopic images submitted during the pilot trial by 1 of their patients, which resulted in an unnecessary clinic visit. Four of the 6 clinicians who recounted experiences assessing dermoscopic images taken by patients or by other clinicians said that assessing lesions from a dermoscopic image (even if high quality) without seeing the patient in person was difficult and not always possible.

And some lesions are just impossible to assess, I think, via telehealth adequately, particularly if patients are high risk, you know…so I think we all feel a bit more comfortable seeing that patient face to face.

Clinician #6, teledermatologist

Similarly, 2 treating clinicians described difficulty making judgments about lesion management from images sent to them by patients, as they were unable to assess the lesion’s texture and how the lesion responds to moving the skin.

Conversely, 2 clinicians said that assessing dermoscopic images taken by patients did not pose a problem for them. One was a treating clinician who, speaking hypothetically, said it would be just like having a patient in the consultation room, on the condition that the images were of good quality and of their own patients. The second clinician, a teledermatologist who had many years of experience in reporting dermoscopic images without seeing the patient clinically, emphasized the importance of adequate clinical history to make recommendations based on dermoscopic images.

#### Possibility of Missing Other Lesions of Concern

Clinicians discussed a hypothetical scenario where patient-led surveillance replaces some routine clinical visits (rather than being implemented in addition to routine visits, as was the case in the pilot RCT). Regarding this scenario, 4 clinicians expressed concern about whether patients were able to correctly identify suspicious lesions or would miss changes in areas of their body that are not easily observable. They were concerned that teledermatology could give patients false reassurance due to their selection of lesions to image, and that this could lead to deferring a clinic visit where a melanoma they were not aware of might have been identified.

…You can do more opportunistic checks and maybe you find something else, while if the patient only sends you a photo of the lesion that is concerning them, maybe that one is nothing but maybe next to it there is a melanoma sitting there.

Clinician #2, treating clinician

#### Potential for Medical Overuse

##### Uncertainty and a Cautious Approach

Among the treating clinicians, 2 explained their experiences of how uncertainty when assessing digital images can result in an overcautious approach, which in turn has the potential to result in medical overuse (ie, unnecessary visits and unnecessary biopsies or excisions) if the clinician is not able to act as the gatekeeper for these.

…Overall, you’re going to be a bit overcautious and you know, overcall things to be on the safe side…when you’re just looking at an image, you actually change your threshold to be more inclusive so that you don’t miss anything…as a teledermatologist, you are going to be more cautious, so normally in your clinical practice…there’s a thing called a number needed to treat, so the number of benign lesions you cut out to find one melanoma, and my average is something like 2.5 to 3, so 2.5 to 3 lesions that I cut out to pick up one melanoma; if I was going off photos and teledermatology, that number may double, it might be 5 or 6 or 7 to 1.

Clinician #3, treating clinician

Because the teledermatologist was not the patient’s treating doctor, they assessed submitted images without information about the patient’s clinical management and sometimes without an image to compare to. Therefore, at times, they advised the patient to make an urgent appointment with their treating doctor for lesions that were already being monitored by the treating doctor. This caused confusion and anxiety for the patient, difficulty for the treating clinician who had to delicately explain the situation, and unnecessary urgently scheduled clinic visits. Treating clinicians also tended to act on the teledermatologist’s report if it suggested that biopsy or excision may be necessary to ease anxiety that the urgent messaging on the report had caused the patient, potentially resulting in unnecessary biopsies and excisions.

…Probably a little bit of anxiety for the patient, thinking oh no there’s something wrong, but because it didn’t take long, they were in [to the clinic] within the week …they were reassured, yep no, this is ok, we’ll do the biopsy anyway, but it should be fine, etc.

Clinician #3, treating clinician

##### Medicolegal Concerns

Four clinicians perceived medicolegal issues associated with teledermatology for management of skin lesions. They suggested that the possibility of litigation for a missed or delayed melanoma diagnosis could be another reason why clinicians may “overcall” a lesion as concerning when it is not, due to the clinician’s tendency to err on the side of caution in their assessment. They explained that providing teledermatology for high-risk melanoma patients should be approached with caution outside a trial context and that some skin specialists prefer not to offer teledermatology for melanoma patients at all.

### Strategies for Achieving High-Value Care

#### Decreasing the Potential for Medical Overuse

##### Adequate Clinical History

All clinicians discussed the difficulty and uncertainty associated with reviewing images “out of context” and stressed the importance of having sufficient lesion and patient history to help them make adequate assessments of dermoscopic images to inform management decisions. Further, they explained that the teledermatologist’s report needs to be considered by the treating doctor as a recommendation; the final decision regarding the management of the lesion is for the treating doctor to make, as they have the most comprehensive knowledge of the patient’s skin.

…You need a history, you need to know where the lesion is, you need to know a little bit about the patient and what the rest of their skin is like. Sometimes what appears to be an abnormal naevus might just be the patient, they actually have all abnormal naevi, and they all look the same, so…there’s not as much concern.

Clinician #5, treating clinician

##### Suitable Patients

To ensure the most effective use of teledermoscopic technologies, clinicians suggested carefully selecting good candidate patients, such as offering them to patients who are at risk of developing a new or recurrent melanoma but only have a small or moderate number of skin lesions to monitor, are comfortable using smartphone apps, have someone to help them take the images, and are interested in actively taking part in their own skin surveillance. Some clinicians also mentioned that patients should be younger (eg, 60 years and under), as these patients are usually more open to monitoring their own skin and are more accustomed to using digital technology. However, this was balanced by the suggestion that exclusion should not be based simply on age because some older people regularly use digital technologies or have someone to help them.

##### Training and Ongoing Support

Providing training, instruction resources, and ongoing support to patients were highlighted as important when delivering a teledermoscopy service. One clinician discussed their experience of patient-performed teledermoscopy in another study:

Our experience has been that when you explain and demonstrate to the patient how to take the photo…and you show them the video. After 3 months, you need to contact them to tell them to look at the video again…so it’s really holding the hand of the patient, and we have quite a lot of phone calls and emails but mostly the videos have been really really useful.

Clinician #1, treating clinician

#### Increasing the Potential for Benefits

##### Replacement of In-Person Monitoring of Specific Lesions

Due to the difficulty of determining with high certainty whether a lesion imaged by a patient is a melanoma or not and the possibility of missing a melanoma, most clinicians felt patient-led teledermoscopy may only partially replace in-person checks. They suggested that patient-led teledermoscopy could be used for patients who would otherwise need to come into the clinic for monitoring particular lesions in between their 6 monthly or annual routinely scheduled in-person checks.

…When we see them, we see some lesions that are not clear-cut melanoma, we don’t want to cut but we are still a little bit worried because it has been changing with the total body photography or the patient has told us it is itchy, but we can’t see anything, there is a lot of good reason to organize monitoring. And so instead of them coming in 3 months afterwards and taking a photo with the photographer and then going home because it’s fine, we just tell them to take the photo at home and send us the photo with MoleScope.

Clinician #1, treating clinician

##### Triage of New Lesions

Clinicians also suggested that patient-led teledermoscopy is well suited for use as a triage tool to decide whether clinical review is necessary when patients identify a lesion they think is suspicious.

So we cannot do a complete check with a dermoscope through Skype or Zoom, but we can triage a lesion quite well, and when we tell them how to send a photo even if they don’t have a dermoscope, most of the time we can tell if we need to intervene now or if it is an age spot that is most likely fine and we review that when they come back in 4 months, or if it’s something that we do need to see because we don’t know it, if it’s too difficult to tell. So, we know how to manage the patient.

Clinician #1, treating clinician

## Discussion

### Principal Findings

This qualitative interview study provides important insights into the experiences and perspectives of a highly specialized group of clinicians (dermatologists and GPs specializing in skin cancer care) who participated in a pilot RCT of patient-led melanoma surveillance using mobile teledermoscopy. Clinicians identified several potential benefits of such an intervention, including additional monitoring and early diagnosis, reassurance for patients, increased access to care, and a reduction in unnecessary clinic visits. Clinicians also discussed several challenges that may increase clinical uncertainty and lead to medical overuse, as well as potentially harming patients. These included receipt of low-quality images from patients (also highlighted by Kozera et al [35]), limitations to assessment of lesions from a dermoscopic image, potential for the patients to miss lesions of concern, and perceived medicolegal risks.

We found that to decrease the potential for medical overuse when implementing patient-led surveillance using teledermoscopy, it is necessary to manage the clinicians’ uncertainty. Uncertainty could be reduced by providing teledermatologists with adequate clinical history, offering the intervention to patients who have been screened for the capacity to use app and photo functions of their smartphone with ease and have someone to help them take dermoscopic photos, and providing patients with sufficient training and ongoing support. For the potential benefits of the intervention to be realized, it is important to ensure its optimum placement within the clinical care pathway. Clinicians suggested that the intervention may be able to replace scheduled monitoring visits for some patients and that the intervention could be used as a triage tool to decide whether in-person assessment is necessary for new lesions. These strategies may ensure that the intervention is most likely to deliver benefits without causing harm.

### Possible Mechanisms and Implications

Although there is potential for the intervention to deliver high-value care, during the pilot trial, several interacting factors resulted in some instances of medical overuse, including unnecessary unscheduled visits and biopsies. Our findings suggest that sometimes teledermatologists (who, as per the trial design, were separate from the treating clinicians) recommended that patients make a fast-tracked unscheduled visit to review lesions that they considered suspicious because they did not have enough information about the lesion or the patient or the image was of poor quality. These decisions may reflect risk aversion, intolerance of uncertainty, and fear of malpractice and litigation, factors that may drive medical overuse [36,37]. In these instances, the treating clinician may have ordered unnecessary biopsies that they otherwise would not have to reassure the patient following an alarming teledermatology report. It is also well known that patients experience significant anxiety concerning their melanoma diagnosis [38], so perceived patient desire for clinical action by clinicians wishing to relieve patient anxiety and prioritize maintenance of the doctor-patient relationship could result in decisions to undertake biopsies even if they think this is clinically unnecessary [39]. Some of these issues may be avoided if patient-led surveillance is implemented with the patient’s treating doctor as the teledermatologist. Where this is not possible, sufficient clinical information needs to be provided to the teledermatolgist.

Underlying the cautious approach taken by teledermatologists and treating clinicians is that patients who participated in the MEL-SELF trial had a personal history of melanoma and were thus at high risk of a subsequent melanoma. The framework by Greenhalgh et al [40] for considering influences on the adoption, scale-up, spread, and sustainability of patient-facing health care technologies considers the nature of the condition as the first domain in theorizing the success or failure of interventions. The diagrammatic depiction of our findings (Figure 1) suggests the ever-present influence of the high-risk nature of melanoma surveillance on clinicians’ perceptions and decisions related to mobile teledermoscopy due to the potential consequences of a missed (or delayed) diagnosis. It follows then that perceived medicolegal risk was high among our sample of clinicians and has been similarly reported in other studies [41], even though perceived risk may be higher than actual risk [42]. Our findings suggest that perceived consequences (for patients and clinicians) of a delayed diagnosis, including medicolegal risk, may have impacted the provision of care. This issue needs to be addressed to minimize the potential for medical overuse from patient-conducted teledermoscopy. Possible solutions might include educating clinicians on the lower-than-perceived actual medicolegal risk, the potential for medicolegal action arising from harm from medical overuse, and the need for transparency about the uncertainty of teledermatology assessment when discussing the process with patients.

Potential harms to the patient from medical overuse such as anxiety, risk of complications from medical procedures, changes to physical appearance, and effects of disease labeling [43] should also be considered by the doctor when making a recommendation via teledermatology or deciding to do a biopsy. Clinicians’ uncertainty associated with patient-performed teledermoscopy may possibly lessen over time as they gain more experience and confidence using the new technologies. A “transition period” after the introduction of new technology, in which clinical management thresholds change and then return to resemble what they were before its introduction, is well documented [44].

### Strengths and Weaknesses

This study is one of the first to explore the experiences of specialized clinicians involved in patient-led surveillance using teledermoscopy. A strength of our study is that our findings are based on unique perspectives from clinicians who had different roles in delivering the patient-led surveillance intervention. Their views reflected their experiences during the MEL-SELF pilot trial, other trials of patient-performed teledermoscopy, and teledermatology in their practice in general. However, accounts of mobile teledermoscopy in a trial context may differ from experiences in clinical practice. In particular, unlike in our trial, the treating clinician may often be the teledermatologist in clinical practice. We also acknowledge that our study sample was small, and our findings are not intended to represent the breadth of experiences and views within each clinician group, namely, skin specialist general practitioners, dermatologists and teledermatologists, and surgical oncologists. A larger sample may have also revealed differences in perspective and opinion between the clinician groups that we did not detect. Additionally, the scope of our inquiry did not allow us to pursue all factors that were previously been found to impact clinicians’ views of teledermatology such as reimbursement, patient privacy, and internet security [7,45]. Finally, the findings of our study are influenced by the health system context in which the clinicians work. Although in Australia, community-based health care receives public subsidy through the Medicare Benefits Scheme, there are still significant out-of-pocket costs, on average AU $183 (US$124) over 6 months for patients in the MEL-SELF pilot trial [46]. All clinics involved in the study are privately operated and charge a fee for service. Moreover, competition between private melanoma clinics exists, but this may not be comparable to the provision of melanoma care in other contexts such as the United States where a broader system of universal health care is not present.

### Future Research

In response to this study’s findings, we made improvements to trial processes for the larger ongoing MEL-SELF trial (ANZCTR12621000176864) [19]. Teledermatologists now have access to an image of the patient’s back to give context on the patient’s skin in general (eg, signs of sun damage), and each patient selects a target lesion with their treating doctor, making explicit the shared monitoring of the lesion. To facilitate monitoring of a lesion, each patient is allocated to 1 teledermatologist who will review all the patient’s images and be able to assess changes over time. Teledermatologists also have the option of seeking a second opinion from another teledermatologist using the DermEngine platform. Each teledermatologist will receive information on subsequent clinical decisions and outcomes as feedback for lesions they have reported on. To encourage perception and use of the intervention as a triage tool, unnecessarily alarming and suggestive terms such as “urgent” or “biopsy” are no longer used in the teledermatologist’s feedback to patients. Further decisions regarding clinical intervention (eg, biopsies, excisions) are left to the treating doctor who reviews the lesion in-person, including the decision to override the teledermatologist’s recommendation. The ongoing MEL-SELF trial is also collecting information on the quality of images taken by patients and whether teledermatologists can report on them.

### Conclusions

This study illuminated the potential benefits and challenges of using patient-performed teledermoscopy from the perspectives of clinicians involved in a pilot RCT of patient-led surveillance. Patient-led surveillance may address inequities in access to melanoma surveillance for patients who live remotely, are less mobile, or require continuity of care during a pandemic [47,48]. It has the potential to deliver high-value care, but safeguards are needed to mitigate against the potential for medical overuse [33,34,36]. The larger MEL-SELF trial, with refinements to the teledermoscopy process in response to the findings from this study, will provide robust evidence on clinical effectiveness to inform the potential wide-scale adoption of patient-led surveillance.

## Appendix

Multimedia Appendix 1 Interview topic guide.

Multimedia Appendix 2 Illustrative quotations.

## Abbreviations

AJCC: American Joint Committee on Cancer
GP: general practitioner
RCT: randomized controlled trial
SRQR: Standards for Reporting Qualitative Research
SSE: skin self-examination
