# Supplementary material for: Perspectives and Experiences of Patient-Led Melanoma Surveillance Using Digital Technologies From Clinicians Involved in the MEL-SELF Pilot Randomized Controlled Trial: Qualitative Interview Study
Source: JMIR Dermatol. 2022 Dec 20;5(4):e40623. doi: 10.2196/40623 (PMC10334935; doi:10.2196/40623)
Supplement: Multimedia Appendix 1 [file derma_v5i4e40623_app1.docx]

# MEL-SELF: A randomised controlled trial of patient-led surveillance compared to clinician-led surveillance in people treated for localised melanoma

## Evaluation of Pilot: Indicative Topic Guide – Clinicians

| KEY AREAS OF INVESTIGATION | EXAMPLE QUESTIONS |
| --- | --- |
| **Introduction** | What are your thoughts regarding patient self-surveillance in general?  What were your initial thoughts regarding patient self-surveillance, using mobile dermoscopy and teledermatology reporting?    How did your thoughts/perceptions change over the course of the trial?  What are your thoughts about the benefits and drawbacks of teledermatology?  Prompt: for rural/urban patients to supplement or replace clinic visits; benefits during a pandemic.  How may the COVID-19 pandemic have influenced your views regarding teledermatology?  How do you feel about assessing photos sent to you by a patient?  What do you think needs to be in place on the patient’s side/what conditions need to be met for use of teledermatology to work?  How confident are you that patients can use the dermatoscope well enough? |
| **Recruitment** | Can you please describe your experience of recruiting patients into the MEL-SELF pilot study?*  What kind of patient would you recruit to the study?  Would you consider recruiting patients for the full MEL-SELF study? If so, what would help you, and do you have any suggestions on the best way to do this in your clinic? |
| **Training/patient support** | If necessary, how could patient training in self-examination, or using the mobile dermatoscope, be improved?  What are your thoughts about the smart phone app and mobile dermatoscope used in the pilot trial in terms of ease of use for patients? |
| **Clinical care**  Experience of intervention for clinician.  Perception of patients’ experience of the intervention. | What kind of conversations did you have regarding the intervention with your patient/s?  How did you integrate use of the intervention into usual patient care?  What kind of benefits did **you** experience as a result of integrating the intervention into care? Why was that?  What were the benefits of the intervention for your patients and/or clinic/health service? Why was that?  If the intervention posed a hinderance to care in any way, could you please describe what and why that was.  What are the potential harms of the intervention for patients and/or the clinic or health service? Why is that?  Which part/s of the intervention or the way it is integrated into care could be improved to mitigate the issues you have described?  What is your impression of your patient’s/s’ general perceptions towards the intervention? Did that seem to change over the course of the pilot trial?  What is your perception of how your patient/s used the smart phone app and dermatoscope?  What kind of obstacles do you think patients encountered and why?  What do you think could help mitigate these issues?  What do you think it was about the intervention (or other components of the trial) that caused your patient/s or other patients to drop out of the trial? |
| **Teledermatology Reporting** | Could you please describe your experience of and, in your opinion, the clinical usefulness of the teledermatology images you reviewed?*  What did you think of the clinical usefulness of the teledermatology reports/images for your patients?* |
| **Implementation** | If you were to adopt and use the intervention in your practise, how would it be implemented within the clinic? In terms of who would conduct the training, and who would follow up on teledermatology reports etc. |
| **Suggestions/conclusion** | Which aspects of the intervention did you find difficult or inconvenient? What do you think could be changed or improved?  Do you have any additional feedback for the MEL-SELF trial team? |
| *if relevant to participant | |
