# Supplementary material for: Perspectives and Experiences of Patient-Led Melanoma Surveillance Using Digital Technologies From Clinicians Involved in the MEL-SELF Pilot Randomized Controlled Trial: Qualitative Interview Study
Source: JMIR Dermatol. 2022 Dec 20;5(4):e40623. doi: 10.2196/40623 (PMC10334935; doi:10.2196/40623)
Supplement: Multimedia Appendix 2 [file derma_v5i4e40623_app2.docx]

| Table 1: Perceived benefits of patient-led surveillance and patient performed teledermoscopy | |
| --- | --- |
| Theme | **Illustrative quotations** |
| Increased and more convenient access | *“…we could see remote patients, patients from far away and that would be fantastic for guys who are elderly or remote NSW and cannot travel to us, so… the opportunity to be more equal for everyone is great.”* (Clin01, treating clinician)  *“Yes, I think that’s a very important benefit for patients who live far away, because I have patients who drive 4-5 hours to come and see me, so if we have something instead, maybe not for every skin check but for some lesions of concern we’re doing, that’s a really great help for them so they don’t have to come all the way here, and I think it will be a really good thing to have for rural patients.”* (Clin02, treating clinician)  *“Often we just take a 3 month, like we might take a photo and then 3 months later ask them to come back in, but if they’re from the country that’s difficult for them, that’s two trips within a 3 month period, so if they’ve sent in the initial photo and we’re like oh it is a little bit unusual but not enough I don’t think and if you’re fairly confident that at that stage its ok just to follow up with 3 month photo self-monitoring, is great, you know, to have that freedom for the patient.”* (Clin08, treating clinician) |
| Reassurance for patients | *“… it’s certainly a worthwhile adjunct to their treatment, it also gave them some reassurance as well that in between their 6 monthly visit or 12 monthly visit, they could have the reassurance that they could have something looked at whenever they wanted to.”* (Clin07, treating clinician)  *“…it’s good for the patient because they have the answer quickly, the answer of what they want to know about, and ah to calm down their anxiety about this lesion that is bleeding or whatever they are not happy with.”* (Clin01, treating clinician) |
| Reduction in unnecessary clinic visits | *“… usually high risk patients they come every 6 months but sometimes they need to come also in between, maybe every 3 months if we find something a little bit suspicious and we don’t want to wait for the full 6 months, so we were giving them the dermoscope so they could send a photo instead of coming… it avoids unnecessary consultations, if we can tell them that it is something looking completely benign then they don’t need to come…”* (Clin02, treating clinician) |
| Early detection | *“… we may pick up an early melanoma, or if it’s something just slightly suspicious, not too bad, then we can tell them to take a photo in three months… and then we’re picking it up at 3 months instead of 6 months, or one year, so we can make an early diagnosis.”* (Clin02, treating clinician) |

| Table 2: Perceived challenges and potential harms of teledermatology and teledermoscopy | |
| --- | --- |
| Theme | **Illustrative quotations** |
| Challenges | |
| Making judgements from a digital image | *“…after the teledermatologist has reviewed the images they sent in, they’ve been asked to come in and when you look at the lesion and the image they’ve taken and you go, well that’s why they’ve been asked to come in, because it is a poor quality dermoscopic image.”* (Clin03, treating clinician)  *“… it is sometimes difficult to tell whether a lesion is benign or a melanoma or just a compound naevus when you’re just seeing it as a two-dimensional image, so you do tend to over call things for review when you’ve just got the dermoscopic image verses when you’ve got the patient there…”* (Clin07, treating clinician)  *“The dermatoscopic photo on itself, sometimes it’s very clear cut what it is, but most times you need, I mean as with everything you need a history, you need to know where the lesion is, you need to know a little bit about the patient and what the rest of their skin is like. Sometimes what appears to be an abnormal naevus might be just the patient right, they are actually all abnormal naevi and they all look the same, so if they all look the same or very similar there’s not as much concern.”* (Clin05, treating clinician) |
| Possibility of missing other lesions of concern | *“If you do the telederm, the question is now which lesions are you imaging?”* (Clin 04, teledermatologist)  *“When you’re examining a patient normally, you’re only going to pick up a lesion if you put the dermatoscope on it, if you don’t put the dermatoscope on half the lesions on the patient’s body, there’s potential that you are missing a lot of things, so if the patient is just submitting several lesions that they’re concerned about but as I said, there’s all these other lesions on the rest of them that they are unaware of, it could be the issue, there is a potential for delayed diagnosis and things being missed, if they’re not having regular follow up that is, as in regular checks and so forth, imagine someone living an 8 hour drive away and the only way they can check is if they submit some images, that’s the only check they’re getting, so there’s an issue for missing things that aren’t being looked at by the patient.”* (Clin03, treating clinician) |
| Potential for medical overuse | |
| Uncertainty and a cautious approach | *“For the doctor it’s a lot of responsibility, with not a perfect set of questions and assessment that we’ve been used to having when we can feel the lesion, when we can ask the patient… so especially for the doctor it is less comfortable.”*  (Clin01, treating clinician)  *“…if you see an abnormal spot as a single photograph, but when you look at the patient and all their other spots look exactly the same, you’re more likely not to biopsy, but if you’re just seeing that one image and you don’t know what the rest of the patient is like then you’re more likely to say go and see your doctor for a biopsy on that spot.”* (Clin03, treating clinician)  *“…so there was a little bit of confusion, because maybe there was a lesion that I had been monitoring for ages, but when the patient takes the photo it went to another doctor and they didn’t have all the background information and maybe didn’t have the comparison so maybe they said that the lesion looks suspicious and we need to excise it but then when I could compare with the photo I could see that it was not changing at all, and then the patient had a bit of conflict like oh this lesion has been monitored for ages but now someone else is saying that it is suspicious…”* (Clin02, treating clinician) |
| Medico-legal concerns | *“You have to have a clinician who is willing to actually say to a patient by seeing just the image, happy to accept that this is fine, so I think medico-legally there has to be a body of work to actually say that it reasonable to do that, I don’t think it’s completely clarified yet, there has been no test case, yet, I am sure it will come, but you know, as a dermatologist I think it would have to be a little bit cautious about doing it and it’s alright in theory for research but actually actioning it, I think there’s a body of work from a medio-legal point of view that has to be done before you will see uptake of it significantly with the dermatologists…”* (Clin05, treating clinician) |

| Table 3: Strategies for ensuring high value care | |
| --- | --- |
| Theme | **Illustrative quotations** |
| Decreasing the potential for medical overuse | |
| Adequate clinical history | *“Having a bit of history about the lesion is really important… is it new or is it changing, and is the patient concerned about it… have they had melanoma in the past, have they had BCCs or SCCs in the past, and you know, sort of basic risk factors are good to have… it is important to know whether there has been any trauma to it too because sometimes they’ve just knocked or scratched something… getting an overview picture of what their skin is like and how much sun damage they have is quite useful…”* (Clin06, teledermatologist)  *“… provide a structured sheet with information, age, sex, ethnicity etc, it makes a difference if you tell me my mother died of melanoma and my aunty”* (Clin04, teledermatologist) |
| Suitable patients | *“The ones who are perfect are dynamic, who are young, who manipulate their phone upside down and who have help at home, it will help someone who really will know what to do.”* (Clin01, treating clinician)  *“…patients who are at higher risk for melanoma, so they’d had a melanoma before… it becomes challenging for patients that’ve got chronic sun damaged skin and get lots of squamous and basal cell skin cancers because often they’re covered in other spots anyway, so the older they are the more benign lesions they have and it starts to complicate things, whereas the younger patients, so someone sort of between 30 and 60, that doesn’t have too many other spots, then it certainly makes it a lot easier to tell if something is significant or not.”* (Clin 07, treating clinician)  *“I think the person needs to have a general confidence with using technology to begin with because they need to be able to attach the dermatoscope to their phone and they need to be able to move their phone… to be able to reach certain parts of their body and take photos, unless of course they have a partner or someone who can help them, so I mean I wouldn’t exclude a person on age, just their ability to negotiate the technology really...”* (Clin 08, treating clinician) |
| Training and on-going support | *“And then they have to be trained or educated about what lesions they need to be looking out for and then they need to be trained to know how to actually take good photographs, so a lot of problems that I’ve seen with patients sending random photographs is that they’re mostly blurry or difficult to see what part of the body they’ve imaged and so on.”* (Clin06, teledermatologist) |
| Increasing potential for benefits | |
| Partial replacement; replacement of in-person monitoring of specific lesion(s) | *“I think it’s something to do in addition of skin checks but not completely substitute the skin checks because if not we are relying only on lesions that are suspicious for the patient, but maybe those are not the important ones, maybe they are just taking photos of the easiest spots and none of them are melanomas that could be there.”* (Clin02, treating clinician)  *“Now in the long run if we stop doing our examination, I think I have a real problem with doing that for melanoma patients, because we are very good at what we are doing, we are detecting melanoma that are less than 4mm, very small little things, I think we could decrease a little bit the number of clinic visits but you know it will be because we can’t see everybody...”* (Clin01, treating clinician) |
| Triage of new lesions identified by the patient | *“… taking the place of a routine surveillance check, it doesn’t do that really, because often times you’re finding things that the patient wasn’t really aware of …for actual diagnosis I think it’s difficult, I think it really is more of a triage tool, does this patient need to be seen next week or can it wait 3 months.” (Clin06, teledermatologist)* |
